# 

**Published:** 1884-12

**Authors:** 


CONTENTS.

| | Page. |
|---|---|
| Editorial Announcement | 219 |
| Cocaine Muriate as a Local Anesthetic | 220 |
| Pine Extract for Bathing | 221 |
| Valuable Remedy for Headache | 222 |
| Stone Cutting of the Ancient Greeks | 223 |
| Caution Concerning Chloroform and Strychnine | 224 |
| Ice Water in Typhoid Fever | 224 |
| Diagnosis of Sciatica | 225 |
| Aryan Views of Doctors | 225 |
| Providence for Drunkards | 227 |
| Ague as a Prophylactic of Phthisis | 227 |
| Don't Fool With Your Eyes | 230 |
| Much Ado About Five Cents | 231 |
| Alum Treatment of Whooping Cough | 232 |
| How to Kill a Craving for Alcohol | 233 |
| Chinese Child Venders | 234 |
| Flavored Melons | 235 |

